# CAUSAL INFERENCE IN PSYCHOPATHOLOGY OF DEPRESSIVE SYMPTOMS IN MIDDLE-AGED AND OLDER ADULTS

**DOI:** 10.1093/geroni/igac059.2678

**Published:** 2022-12-20

**Authors:** Zheng Zhu, Xiang Qi, Yaolin Pei, Jing Wang, Bei Wu

**Affiliations:** New York University, New York , New York, United States; New York University, New York , New York, United States; New York University, New York , New York, United States; The University of North Carolina at Chapel Hill, Chapel Hill, North Carolina, United States; New York University, New York , New York, United States

## Abstract

Most previous research studied depression as a holistic conception and ignored the complex relationships between depressive symptoms. How depressive symptoms interact with each other is still unknown. The aims of this study were to develop symptom networks of middle-aged and older adults and to explore the core symptom in the symptom networks. This study used three-wave data from the China Health and Retirement Longitudinal Study (CHARLS) in 2013 (T1), 2015 (T2), and 2018 (T3). Depressive symptoms were measured by the 10-item Epidemiological Research Center for Depression Scale (CES-D). A multilevel vector autoregression model (VAR) was used to identify 10 depressive symptoms dynamically interacting with each other over time. A total of 3558 participants were included in the final analysis. The strongest direct effects were “D10: felt fearful” -> “D6: felt everything I did was an effort” (β=0.14). “D10: felt fearful” reported the largest value of out-predictability (r=0.064) and out-strength (r=0.635). “D3: felt depressed” reported the largest value of in-predictability (r=0.077) and in-strength (r=0.545). Substantial heterogeneity in the network may stem from an individual’s gender and living region. “Felt fearful” was the strongest predictor compared to the other nine depressive symptoms based on node centrality. It was also the most crucial bridge node between negative symptoms cluster and positive affects cluster. Network density and the sum of all absolute strength centrality should also be incorporated into clinical practice as key indicators of emotional vulnerability, particularly in male middle-aged and older adults.

